# Cross-Cultural Administration of an Odor Discrimination Test

**DOI:** 10.1007/s12078-014-9169-0

**Published:** 2014-04-22

**Authors:** Agnieszka Sorokowska, Piotr Sorokowski, Thomas Hummel

**Affiliations:** 1 Institute of Psychology, University of Wroclaw, ul. Dawida 1, 50-527 Wroclaw, Poland; 2 TU Dresden, Medical Faculty Carl Gustav Carus, Department of Otorhinolaryngology, Smell & Taste Clinic, Dresden, Germany

**Keywords:** Cross-cultural studies, Discrimination test, Sniffin’ Sticks, Tsimane’

## Abstract

Olfactory sensitivity can be evaluated by various tests, with “Sniffin’ Sticks” test (SST) being one of the most popular. SST consists of tests for odor threshold, discrimination, and identification. It seems relatively straightforward to administer threshold tests in different groups and societies and it has been shown that odor identification tests requires special adaptation before they can be administered to various populations. However, few studies have investigated the application of an odor discrimination task in various regions/cultures. In the present study, we compared the discrimination scores of 169 Polish people with the scores of 99 Tsimane’, Bolivian Amerindians. The Tsimane’ participants scored very low in the discrimination task, despite their general high olfactory sensitivity. This result suggests that when a discrimination task is chosen as the form of olfactory testing, some additional variables need to be controlled. We suggest three sources of low scores of our participants—their cognitive profile, the cultural background, i.e., little knowledge of the odors used in the discrimination test and problems associated with testing environment.

## Introduction

Smell is an important human sense —odors can influence our mood, cognition, and behavior (for a review, see: Herz 2002). The functions of our smell may be categorized into three main groups (Stevenson 2010): related to ingestive behavior (Murphy 1985; Porter et al. 2006), alarm functions—avoiding environmental hazards (Van den Bergh 1999), and functions related to social communication (Ackerl et al. 2002; Sorokowska et al. 2012). Generally, olfaction allows us to detect subtle changes in our environment, but sensitivity of this sense varies across individuals (Murphy et al. 2003).

Olfactory sensitivity can be evaluated by various tests (overview: Thomas-Danguin et al. 2003), with “Sniffin’ Sticks” test (SST; Burghart Messtechnik, Wedel, Germany; Hummel et al. 1997; Hummel et al. 2007; Kobal et al. 1996) being one of the most popular. Previous work has established its test-retest reliability and validity (Kobal et al. 2000) and the use of this test is recommended by the “Working Group Olfaction and Gustation” of the German Society for Otorhinolaryngology, Head and Neck Surgery. The normative data of the SST has been established on the basis of results obtained in thousands of healthy subjects in Europe and Australia (Hummel et al. 2007; Katotomichelakis et al. 2007). SST consists of tests for odor threshold, discrimination, and identification. The composite score of olfactory threshold (OT), odor discrimination (OD), and odor identification (OI) are calculated as the total threshold-discrimination-identification (TDI) score, which is ≤15 for anosmia, ≥30 for normosmia, and in between for hyposmia (Hummel et al. 2007).

The “Sniffin' Sticks” olfactory test has been developed in Europe and is used for assessment of olfactory function in many countries. While it seems relatively straightforward to administer threshold tests in different groups and societies (Hoshika 2006; Sorokowska et al. 2013), it is not as clear whether the two remaining parts of SST—identification and discrimination tests—are equally appropriate for cross-cultural comparisons.

Application of the identification test appears to be difficult in cross-cultural studies. Performance in odor identification test relies on prior exposure to and familiarity with the presented odors (Goldman and Seamon 1992; Richardson and Zucco 1989), so prior to usage of identification tests in a specific culture odors need to be adapted to the subjects’ cultural background (Konstantinidis et al. 2008; Shu et al. 2007). Additionally, odor identification is a semantic memory task and studies show positive relationships among general semantic knowledge, verbal fluency, and proficiency in this test (Hedner et al. 2010; Larsson et al. 2000; Larsson et al. 2004). These two factors could severely limit identification tests’ validity in different cultures or populations with very differentiated levels of general semantic knowledge (for example, in societies where some people do not have access to formal education). Additionally, odor identification can only be used if the test is based on a multiple-choice procedure (Cain and Krause 1979) and the verbal descriptors of odors should also be analyzed (and adapted) before it can be administered. An odor identification task is an important tool for the clinical evaluation of olfactory sensitivity and is generally used in large majority of available olfactory tests (Thomas-Danguin et al. 2003), but the results of identification tests seem to be culture-dependent (Doty et al. 1985; Thomas-Danguin et al. 2001). Thus, it seems that this element of the SST cannot be used for direct comparisons of olfactory sensitivity between different cultures and societies.

SST discrimination task involves a triple-forced-choice procedure. However, unlike the threshold test, all presented pens contain odorants. Per triplet, two distracter pens encompass identical smells, while the respective third pen (the clue) contains a different odor. The number of correctly identified clues represents the discrimination score. Odor discrimination is easier to administer than threshold measurement, and seems to be less language-dependent than is identification. However, Thomas-Danguin and collaborators (2001) showed that, even if odor discrimination seems to be a nonverbal task (Hummel et al. 1997), it is to some extent dependent on culture, probably via familiarity effects.

There exists also another problem, which seems even more important because it might influence the applicability of the SST discrimination test even in one culture of dramatically differing levels of education of the society. In the assessment of odor discrimination abilities, the participant is required to detect similarities and differences between odorants, or different concentrations of a presented odorant (Engen 1986). Thus, discrimination requires that the participants are very concentrated and use their memory and other cognitive abilities. In Hedner et al. (2010) study, the cognitive block (executive function and semantic memory composites) accounted for a significant portion of the variance (11.5 %) in odor discrimination. Basically, variations in cognitive function (and thus the performance in discrimination task) are inevitable in the general population. But generally, populations differ in terms of development and education (Human Development Report, 2010) and sometimes, even within a single population, the discrepancy between education level (and related training of higher cognitive functions) of different groups might be high. For example, many more women are illiterate in rural areas of India than in urban areas of this country (69.8 % vs. 39.4 %; Second Human Development Report of State of India 2007). Therefore, it is not clear whether the discrimination test can be used in all groups and populations with equal effectiveness, how well it is suited for cross-cultural comparisons, and which additional variables should be controlled to obtain the least biased olfactory test score of an individual.

In summary, it has been shown that odor identification tests require special adaptation before they can be administered in a various populations; however, few existing studies have analyzed the problems associated with application of an odor discrimination task in various regions/cultures. In the present study, we wanted to test the discrimination performance of a population with no knowledge of odors used in the discrimination test and without “training” in cognitive abilities (i.e., with low education). We compared the discrimination scores of a group of Polish people with the scores of the Tsimane’, Bolivian Amerindians. We chose this population because the Tsimane’ were found to have very good sense of smell (as tested by SST threshold subtest; Sorokowska et al. 2013), but they have limited access to scented cosmetics and modern chemicals. Also, most Tsimane’ adults are illiterate (Godoy et al. 2010) and a large percentage of Tsimane’ does not receive any formal education (Godoy et al. 2005; Kirby et al. 2002; Reyes-Garcia et al. 2007).

## Materials and Methods

### Participants

The first phase of the study tested 99 Tsimane’: 53 females aged 18–51 years (*M* = 29.51, SD = 9.44) and 46 men aged 18–50 years (*M* = 32.13 years, SD = 10.90) from six villages along the Maniqui River. None of them reported otorhinolaryngological problems at the time of the study. They received a gift (household items worth ∼6 USD) for participating in a series of studies. The second part tested 169 Polish people from Wroclaw: 94 females aged 19–60 years (*M* = 30.47, SD = 12.16) and 75 males aged 18–60 years (*M* = 31.25, SD = 12.52).

The study was conducted according to the principles expressed in the Declaration of Helsinki. The study protocol and consent procedure received ethical approval from the Institutional Review Board (IRB) of the University of Wroclaw (Wrocław, Poland) and from the Great Tsimane’ Council (the governing body of the Tsimane’). All participants provided informed consent before study inclusion. Due to the low levels of literacy of the Tsimane’, we obtained oral consent for participation and documented it using a portable recorder.

### Procedure

Trained experimenters assessed olfactory function of participants using the “Sniffin’ Sticks” discrimination subtest (Burghart Messtechnik, Wedel, Germany). A translator explained the procedure to the participants in their native languages. We ensured that the Tsimane’ participants understood the procedure before completing the task. That is, first, we explained to them that they will smell a set of three pens out of which two contain the same smell and one is different. We gave an example from their everyday life—like that one pen could smell with soap and two could smell of food and they should show us which pen is different. We asked them to smell a randomly chosen set of three pens, and when they confirmed that they had smelled all of them, we asked them to choose the differently smelling pen from the set. If they performed the task correctly (they pointed the pen of different smell or—alternatively—they showed which two pens had the same odor) we continued with the standard procedure. If they did not perform the task correctly, we explained the procedure again and asked them to smell the same three pens one by one, this time showing them the correct answer and asking whether they can smell the difference. When they confirmed and said that they understood the task after this additional explanation, we continued with the standard procedure. However, some participants did not understand the task. They were expressing it in many different ways—for example, some said that the test was “stupid” or “difficult,” and some stopped answering the questions. We excluded these participants from further participation. Still, a few Tsimane’ participants who took part in the test admitted that they had been guessing the answers.

## Results

The results of the Tsimane’ (Shapiro–Wilk *W* = .971, *p* = .03) and Polish participants (Shapiro–Wilk *W* = .927, *p* < .001) were not normally distributed. Therefore, we presented the results for the two groups as medians and interquartile ranges and compared them using the non-parametric Mann–Whitney *U* test. We undertook two-tailed tests throughout, using STATISTICA ver 10 (StatSoft, Inc.) with *p* < .05 as the level of significance.

For Tsimane, the percentages of correctly discriminated triples ranged from 35 to 57 %, with an average of 47 %. For Polish people, the percentages ranged from 41 % to 91 %, with an average 74 %. Median values in the Tsimane’ and Polish groups were 7 and 13, respectively. The lowest result in the Tsimane’ group was 1 and the lowest result in the Polish group was 5; the highest results were 14 and 16, respectively. Interquartile range in Polish group was 3 (with *Q*1 = 11 and *Q*3 = 14) and in Tsimane group 5 (with *Q*1 = 5 and *Q*3 = 10).

Subjects from the Tsimane’ group performed in the test significantly worse than did Polish subjects (Mann–Whitney *U* test *U* = 1,249_169,99_, *p* < 0.001, *Z =* 11.62). The scores for males and females did not differ significantly in the Polish (*U* = 3,503.5_94,75 ,_ *p* = .95) or Tsimane’ groups (*U* = 1,186.5_53,46_, *p* = .82).

Results were also analyzed using an analysis of variance for repeated measures (rm-ANOVA; program package SPSS 21.0 from SPSS Inc., Chicago, IL., USA) with “test” as within subject factor (odor threshold, odor discrimination) and “group” as between subject factor (Tsimane, Polish). Although there was no significant effect of the factor “test” (*F*[1,309] = 0.58, *p* = 0.45), overall, Polish subjects scored higher than Tsimane subjects (factor “group” *F*[1,309] = 39.7, *p* < 0.001). However, as indicated by the interaction between factors “test” and “group” (*F*[1,309] = 179.9, *p* < 0.001) for odor thresholds, Tsimane subjects scored higher than Polish subjects while this was the other way around for odor discrimination.

## Discussion

The Sniffin’ Sticks test battery has been used in a large number of studies, and is a part of the everyday rhinological clinical practice in many countries (Hummel et al. 2007; Kobal et al. 1996; Konstantinidis et al. 2008). In our study, we analyzed the results of the SST discrimination subtest in two cultures. Threshold subtest of SST seems to be suitable for cross-cultural and cross-regional comparisons (Sorokowska et al. 2013), whereas odor identification tests typically need to be adapted for application in various cultures/regions (Thomas-Danguin et al. 2001). However, odor discrimination tests have not been analyzed from this perspective before. The Tsimane’ participants who took part in our study scored very low in the discrimination task, despite their high olfactory sensitivity (Sorokowska et al. 2013). This result suggests that when discrimination task is chosen as the form of olfactory testing, some additional variables need to be controlled. We suggest three sources of low scores of our participants—their cognitive profile, the cultural background, i.e., little knowledge of the odors used in the discrimination test and problems associated with testing environment.

Odor discrimination involves complex processing of olfactory information. Hedner and collaborators (2010) showed that odor discrimination/identification was significantly connected to cognitive proficiency—participants who performed well in executive functioning also discriminated and identified more odors correctly. Also, Larsson (1997) and Larsson and collaborators (2004) showed that proficiency in general knowledge and tasks like letter fluency or vocabulary was positively related to discrimination. These reports suggest that individual’s cognitive profile exerts a significant influence on higher order olfactory performance (Dulay et al. 2008; Hedner et al. 2010; Larsson et al. 2004). Interestingly, in the same study, no such influence was observed for the olfactory threshold test (Hedner et al. 2010). It is then possible that the cognitive load necessary to complete the discrimination task might be too high for people who did not have access to formal education, even if their general olfactory sensitivity (assessed by threshold test, like in Sorokowska et al. 2013) is high.

The second source of difficulties could be low knowledge of the odorants used in the discrimination test. Olfactory sensation is a complex biocultural process, and it is not a passive, merely receptive enterprise (Shepard 2004). In his work, exploring the nascent field of “sensory ecology,” Shepard (2004) defined a new theoretical perspective, in which sensations are rooted in human physiology, but also constructed through individual experiences and culture. Organoleptic properties can change over time and across and between different cultures, because inherent qualities of chemical substances appear to interact with individual experience, context, and, to a large extent, cultural conditioning (Doty 1986; Wysocki et al. 1991; Shepard 2004). Olfaction plays important roles in (among others) dietary habits, religious beliefs, medicine, memory, and sexuality of various societies (Classen 1992; Gollin 2004; Jernigan 2008; Leonti & Sticher, 2002; Shepard 2004; Sorokowska 2013), but these roles and their importance might differ across cultures. For example, Pieroni and Torry (2007) showed that links between taste perceptions and the medicinal uses of herbal drugs may be quite different across diverse cultural groups (South-Asians (Kashmiris and Gujaratis) and English people), and Majid and Burenhult (2014) showed that some cultures (like Jahai, Peninsular Malaysia) find it easier to name odors than others (native English speakers). All the aforementioned findings are important for the interpretation of our results. Discrimination score might depend on the familiarity of the odors (Thomas-Danguin et al. 2001). Tsimane’ knew smells of some of the objects used in the test—like smell of a banana or camphor—but they rather did not have (frequent) contact with the artificial equivalents—like isoamyl acetate and fenchone. It is possible that for societies that are weakly industrialized and/or have rare contact with chemically created smelling substances, the discrimination test might be too difficult. Given that (while firmly rooted in physiology), sensation is also shaped by individual experience, cultural preconditioning, and environmental variables (Shepard 2004), the solution for similar, future research programs might be to work ethnographically on local odor categories—in terms of classification, real-world referents and symbolic and practical meanings—in conjunction with any comparative scientific methods. Perhaps, local people (unaccustomed to encountering odors in pure synthetic form) need other cues—the actual odorous plant leaf, or fruit, or flower, or object—to better discriminate odors. Such custom-made, culturally-adapted tests would enable the scientists to analyze actual olfactory abilities of people from all over the world. So far, the general olfactory assumptions seem to be based upon tests of the people from “WEIRD” (Western Educated Industrialized Rich Democratic; Henrich et al. 2010) countries, which makes universality of these findings questionable.

There exist also other reasons of lower performance of the Tsimane’. Testing among Tsimane involved some “environmental” problems—the houses where we conducted our study did not have solid walls, so the participants could hear a lot of noise from the villages—like animal sounds, voices of other people, etc. Recently, Seo et al. (2011) showed that subjects’ performance in the odor discrimination task was impaired in the presence of background noise. The authors explained that as the odor discrimination task is highly dependent on cognitive ability and education level (Boesveldt et al. 2008; Hedner et al. 2010), the noise-induced deteriorated performance could be mediated by the interruption of cognitive processes required to perform the task (Seo et al. 2011). However, the average Tsimane result was so much lower than European that probably this discrepancy was not simply the effect of background noise alone.

## Conclusions

Olfactory tests have significantly increased the understanding of the sense of smell in humans (review by Doty 2001) and cross-cultural testing is an exciting area of olfactory studies. However, few existing studies have analyzed the cross-cultural applicability of tests other than identification. Our study shows that there are some problems associated with exploitation of the discrimination test and that researchers should be aware of possible cultural and social differences which may cause different performances in this olfactory test.
